# 19-Hydroxybufalin inhibits non-small cell lung cancer cell proliferation and promotes cell apoptosis via the Wnt/β-catenin pathway

**DOI:** 10.1186/s40164-021-00243-0

**Published:** 2021-10-25

**Authors:** Wei Yu, Xiao Zhang, Wei Zhang, Minggang Xiong, Yuhan Lin, Ming Chang, Lin Xu, Yi Lu, Yun Liu, Jian Zhang

**Affiliations:** 1 Guizhou Provincial College-Based Key Lab for Tumor Prevention and Treatment With Distinctive Medicines, Zunyi Medical University, Zunyi, 563000 China; 2 School of Medicine, Southern University of Science and Technology, Shenzhen, 518055 Guangdong China; 3 Guangdong Provincial Key Laboratory of Cell Microenvironment and Disease Research, Shenzhen, 518055 Guangdong China

**Keywords:** 19-Hydroxybufalin, Apoptosis, NSCLC, Migration and invasion

## Abstract

**Background:**

Bufadienolides derived from the skin of toads are often regarded as the main active components with antitumor effects. 19-Hydroxybufalin (19-HB) is a monomer of bufadienolides; however, its effects and underlying molecular mechanisms on tumor growth remain to be ascertained. In this report, we focused on the antitumor effects of 19-HB on non-small cell lung cancer to provide a scientific basis for its further development and utilization.

**Methods:**

The antitumor effects of 19-HB on the human NSCLC cell lines NCI-H1299 and NCI-H838 were examined in vitro*.* The cells were treated with different concentrations of 19-HB, and the inhibition of cell growth was measured by CCK-8 and colony formation assays. Furthermore, cell apoptosis was analyzed by flow cytometry, TUNEL staining, JC-1 staining, and western blotting. The effects on migration and invasion were evaluated by wound-healing assay, transwell assay, and western blotting. Finally, the antitumor effects of 19-HB were evaluated in vivo using a xenograft mouse model.

**Results:**

19-HB-treated NSCLC cells showed inhibited cell viability and increased apoptosis. The expression levels of cleaved caspase-3, cleaved-PARP, and Bax/Bcl-2 were upregulated, while the mitochondrial membrane potential decreased. In contrast, migration, invasion, as well as the expression of MMP2, MMP7, MMP9, the epithelial–mesenchymal transition-related proteins N-cadherin and Vimentin, and the transcription factors Snail and Slug were inhibited. Furthermore, the expression levels of the key molecules in the Wnt/β-catenin signaling pathway (CyclinD1, c-Myc, and β-catenin) were decreased. In vivo*,* the growth of xenograft tumors in nude mice was also significantly inhibited by 19-HB, and there were no significant changes in biochemical indicators of hepatic and renal function.

**Conclusions:**

19-HB inhibited the proliferation, migration, and invasion, and promoted the apoptosis of NSCLC cells via the Wnt/β-catenin pathway. In addition, 19-HB inhibited the growth of xenograft tumors in nude mice with little toxicity to the liver and kidney. Thus, 19-HB may be a potential antitumor agent for treating NSCLC.

**Supplementary Information:**

The online version contains supplementary material available at 10.1186/s40164-021-00243-0.

## Background

Lung cancer ranks as the second most common cancer worldwide [1]. Among all lung cancer patients, approximately 85% of them suffers in non-small cell lung cancer (NSCLC) based on histological characters [2]. In NSCLC, orchestrated signaling pathways cooperatively induce tumorigenesis, progression, and poor outcomes. Surgery, radiation therapy, chemotherapy, and targeted therapy are common current treatment options for NSCLC. Recently, immunotherapy has significantly improved the survival of patients with NSCLC or small cell lung cancer (SCLC) [3–5]. However, majority of lung cancer patients are not benefit from immunotherapy [6]. Currently, these options only provide limited effects on the overall survival, particularly in individuals with metastatic and chemoresistant phenotype. Therefore, searching for new molecular target and high-efficiency drugs is urgently needed [7–9].

**Fig. 1:**
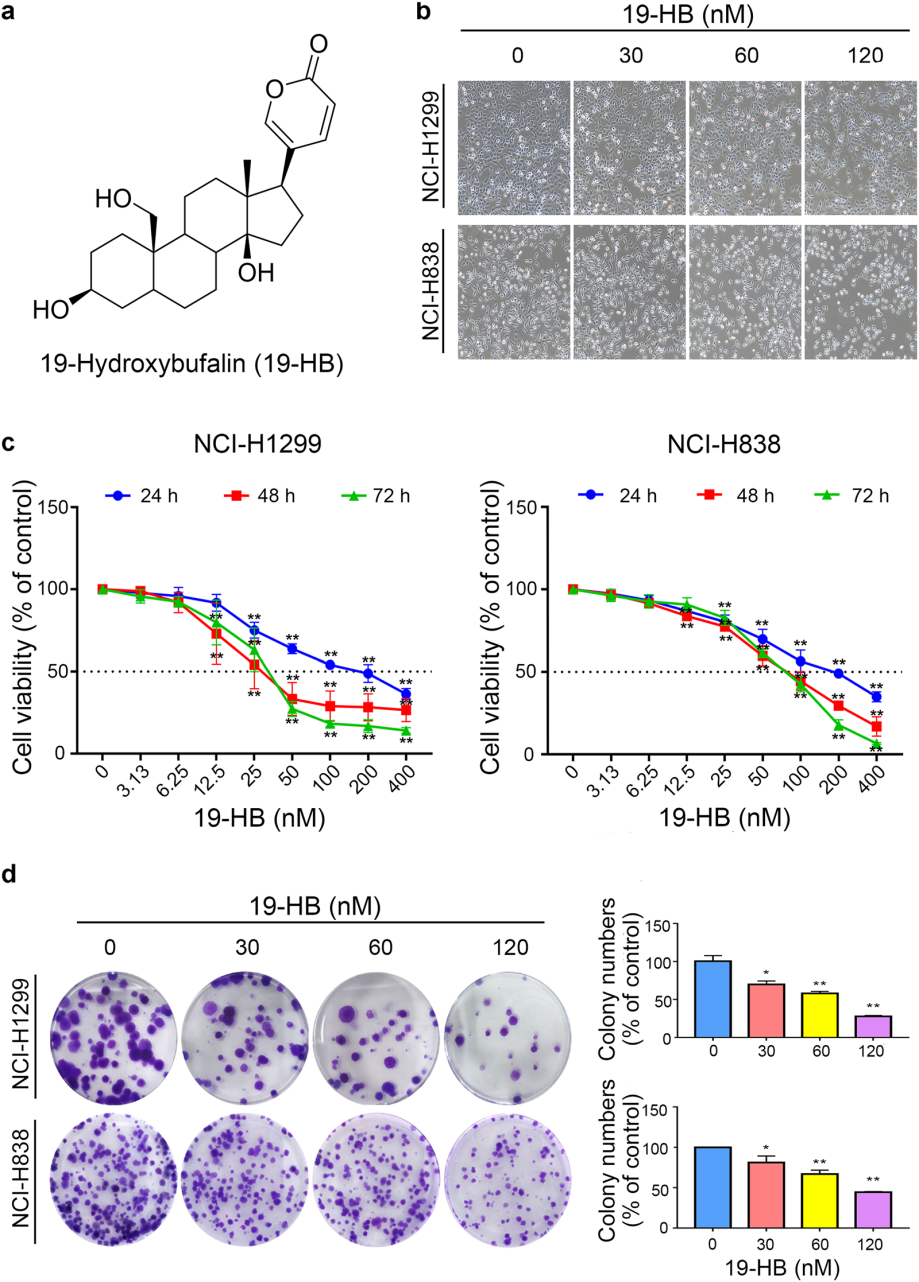
19-HB inhibited proliferation and changed cell morphology in NSCLC cells. **a** Chemical structure of 19-HB. **b** Light scope showed cell morphology changes in NSCLC cells treated with 19-HB for 24 h. **c** Cell viability was determined by a CCK-8 assay at 24, 48, and 72 h after treatment. **d** Cell proliferation determined by colony formation assay and colony formation numbers were calculated after 14 days of culture in 24-h 19-HB-treated cell lines. The data are presented as the mean ± SD of three parallel tests (*P < 0.05 vs. control, **P < 0.01 vs. control)

Some natural products originating from Traditional Chinese Medicine (TCM) exhibit potential antitumor activities and have been used for the treatment of malignant tumors in China. An illustrative example is *Bufo bufo gargarizans Cantor*; it is an extremely precious medical material in China, well known as a TCM due to its pharmaceutical value [10]. Toad glandular secretions (“chansu” in Chinese), skin extractions, as well as the dried skin (“chanpi” in Chinese) have traditionally been used in cancer treatment [11]. These ingredients have been shown to exert antitumor effects, such as antiproliferative, proapoptotic, and antimetastatic effects; moreover, they regulate immunocompetence, reverse multidrug resistance, and attenuate cancer-derived pain [12–14]. The Huachansu (Cinobufacini) and Chansu injections have also been used in clinics with promising therapeutic effects in treating colorectal cancer, lung cancer, liver cancer, and other tumors as well [15–17]. Recent studies suggested that bufadienolides were important components of chansu and chanpi and might attribute to the bioactivity of them. 19-Hydroxybufalin (19-HB, Fig. 1a) [18], an ingredient of bufadienolides from the skin of toads, might participate in the antitumor activities. However, there is still no evidence of its effects and the potential biological mechanism for the application in lung cancer.

To explore the value of 19-HB in treating lung cancer such as NSCLC, we investigated the effects and toxicity of 19-HB on NSCLC and explored the potential molecular mechanism. We found that 19-HB inhibited tumor progression through inhibiting the cell viability of NSCLC cells and promoted cell apoptosis. During the process, β-catenin, the key factor in Wnt/β-catenin signaling pathway, as well as two classical dowmstream target genes, c-Myc and CyclinD1, were significantly decreased, suggesting that the antitumor effects of 19-HB may be mediated through suppressing Wnt/β-catenin signaling pathway. In addition, biochemical indicators of hepatic and renal function showed that 19-HB also caused less hepatorenal toxicity. Our study provided a scientific basis for the clinical development and utilization of 19-HB, including further combinations with other antitumor therapies.

## Materials and methods

### Reagents

19-HB was prepared from an aqueous extract of Chanpi and was prepared as a stock solution of 10 mM in DMSO. Western blot antibodies targeting Bcl-2, Bax, cleaved-caspase3, total PARP, cleaved-PARP, MMP2, MMP7, MMP9, c-Myc, N‐cadherin, β-catenin, Vimentin, Snail, Slug, and Cyclin D1 were purchased from Cell Signaling Technology (Beverly, MA, USA), and the antibody against α-Tubulin was bought from Proteintech (Rosemont, IL, USA).

### Cell culture

Human NCI-H1299, NCI-H838, A549, Huh7, HepG2 and MDA-MB-231 cell lines were obtained from the American Type Culture Collection (Manassas, VA, USA). The cells were cultured in RPMI 1640 or DMEM medium with 10% fetal bovine serum (FBS) and supplemented with 1% penicillin and streptomycin (Invitrogen, Carlsbad, CA, USA). The cells were maintained in a humidified chamber at 37 °C containing 5% CO_2_. Cell morphology was observed under an inverted microscope (ECLIPSE Ti2, Nikon Corporation, Japan).

### Cell viability assay

The cytotoxic effects of 19-HB on the cancer cell lines NCI-H1299, NCI-H838, A549, Huh7, HepG2 and MDA-MB-231 were determined by Cell Counting Kit-8 (CCK-8) assay (Yeasen, Shanghai, China). The cells were suspended in the medium described above that contained 10% fetal bovine serum (FBS) and antibiotics, then seeded in 96-well plates. Twenty-four hours later, different concentrations of 19-HB were added to the plate and incubated for 24, 48, and 72 h. At each indicated time point, 10 μL CCK-8 reagent was added to the each well of the plate and the optical density (OD) values were measured through the absorbance at 450 nm to calculate the cell viability.

### Colony formation assay

NCI-H1299 and NCI-H838 cells were seeded in 6-well plates (500 cells/well). The cell culture medium was removed 24 h later; the cells were exposed to various concentrations of 19-HB (0, 30, 60, and 120 nM) for 14 days at suitable conditions. At indicated time point, the medium was removed, and the cells were washed with phosphate-buffered saline (PBS) twice. For fixation, the cells were incubated in 4% paraformaldehyde for 20 min, then washed twice with PBS, and stained with 0.1% crystal violet for 20 min. Photographs were taken after washing with PBS to capture clear images, and colonies were counted.

### Flow cytometry analysis

The apoptosis process induced by 19-HB was detected through a fluorescein isothiocyanate (FITC) Annexin V Apoptosis Detection Kit I (BD, Franklin Lakes, NJ, USA) in accordance with the instrument. In brief, approximately 10^6^ cells that had been treated with specific 19-HB concentration were digested and incubated in 100 μL tubes with binding buffer containing 5 μL of PI and 5 µL FITC-annexin V in dark for 15 min at room temperature. Next, 400 µL of 1X binding buffer was added to each tube, and the double staining signals were finally analyzed through a flow cytometer.

### TUNEL (TdT-mediated dUTP nick-end labeling) staining

TUNEL assay (Beyotime, Shanghai, China) was used to examine the DNA fragmentation. The NSCLC cells were seeded onto 6-well plates and cultured for 24 h; then, the cells were treated with increasing concentrations of 19-HB (0, 30, 60, and 120 nM) for totally 24 h. The cells were fixed with 4% paraformaldehyde and incubated in accordance with the manufacturer’s instructions before being observed under a fluorescence microscope.

### Mitochondrial membrane potential assay

The NSCLC cells were exposed to 19-HB (0, 30, 60, and 120 nM) for 24 h. According to the instructions, the cells were centrifuged, resuspended, and incubated in JC-1 staining solution. Finally, the JC-1 staining signal was analyzed by flow cytometry.

### Western blot analysis

Whole-cell protein extracts were prepared with RIPA and protease inhibitors (99:1). Proteins were separated by sulfate-polyacrylamide gel electrophoresis and transferred to polyvinylidene difluoride membranes by electrotransfer. The membranes were blocked with 5% BSA in Tris-buffered saline with Tween-20 and subsequently incubated with primary and secondary antibodies. The proteins on the membranes were visualized using the Chemiluminescent Reagents Kit. Chemiluminescent signals were detected with ChampChemi and quantified using ImageJ.

### Transwell assay

NCI-H838 and NCI-H1299 cells (2 × 10^4^ cells/100 μL) were added to serum-free medium to the transwell upper chamber (Corning Star; Cambridge, MA, USA) with or without Matrigel. The indicated concentrations of 19-HB or DMSO were added to the culture medium. Meanwhile, 750 μL medium containing 10% or 20% FBS was added to the lower chamber as a chemoattractant to allow the cells to migrate or invade for 24 h. The migrated or invasive cells on the chamber bottom were stained with 0.1% crystal violet after fixation with 4% paraformaldehyde for visualization. Five fields per chamber were randomly photographed for the quantification of migrated cells (ECLIPSE Ti2, Nikon Corporation, Japan). Invasive index was calculated as the ratio of the number of cells that had penetrated the Matrigel to the number of cells that had penetrated the uncoated Matrigel.

### Wound-healing assay

NCI-H838 and NCI-H1299 cells (2 × 10^4^ cells/well) were seeded into 6-well plates with complete RPMI-1640 medium and allowed to grow until the cell confluence reached > 95%. And a sterile 200 μL pipette tip was utilized to scrap a vertical wound. The cells were later treated with 19-HB (0, 30, 60, and 120 nM) in serum-free RPMI-1640 medium. The same field at each well was imaged with a microscope (ECLIPSE Ti2, Nikon Corporation, Japan) at three time points (0, 12, and 24 h) after the treatments. The images were processed and analyzed using ImageJ software, and the scratch areas of each observation point at different times were measured to calculate the cell migration rate.

### Reverse transcription quantitative real-time PCR

TRIzol (Invitrogen) was used to extract total RNA from cells; the concentration and purity were determined using a NanoDrop spectrophotometer, and RNA was reverse-transcribed using Vazyme Reverse Transcription Reagents. Quantitative PCR was performed on a 7500 PCR machine (Applied Biosystems, Waltham, MA, USA) using SYBR Green PCR Master Mix. The relative gene expression level was calculated using the 2^−ΔΔCt^ method normalized to β-actin. The primer sequences were as follows: forward primer sequence 5′‐AGACATACATCTTTGCTGGAGACA‐3′ and reverse primer sequence 5′-CTTGAAGAAGTAGCTGTGACCG-3′ for MMP2; forward primer sequence 5′-GGAGGAGATGCTCACTTCGAT-3′ and reverse primer sequence 5′-AGGAATGTCCCATACCCAAAGA-3′ for MMP7; forward primer sequence 5′-GGGACGCAGACATCGTCATC-3′ and reverse primer sequence 5′-TCGTCATCGTCGAAATGGGC-3′ for MMP9; forward primer sequence 5′-CATGTACGTTGCTATCCAGGC-3′ and reverse primer sequence 5′-CTCCTTAATGTCACGCACGAT-3′ for β-actin.

### Tumor xenografts model in nude mice

The male Balb/c nude mice (4–6 weeks old) were kept in the Laboratory Animal Center at Southern University of Science and Technology. NCI-H1299 cells (1 × 10^7^ cells in 0.1 mL of PBS mixed with Matrigel) were injected subcutaneously as indicated for nude mice. When the tumor volumes reached 50–80 mm^3^, the nude mice were randomly divided into a control group and a 19-HB group, with 5 mice in each group. The injection concentration of 19-HB group was 1 mg/kg; the control group received normal saline with the same amount of DMSO, and the intraperitoneal injection was given every other day and continued for 16 days. The tumor volume was calculated according to the following formula: volume = width^2^ × length/2, and the tumor growth curve was presented. All the animals were sacrificed by dislocation after anesthesia. The tumor was dissected out from each mouse and the weight of each tumors were measured. The serum of mice was collected for biochemical analysis, and the tumors were collected for further analysis by immunohistochemistry and TUNEL staining.

### Immunohistochemistry and TUNEL staining

Formalin-fixed, paraffin-embedded sections of xenograft tumors were subjected to immunohistochemistry following the routine protocols. The Ki67-positive cells in tumor tissues were counted, and the average number of positive cancer cells was determined from three separate areas in each section of five independent tumor samples. The apoptotic cells in the xenograft tumors from the nude mice were detected by TUNEL staining.

### Statistical analysis

The independent and quantitative data among four groups were evaluated using one-way analysis of variance (ANOVA) followed by Dunn’s post-hoc tests. *P*-values < 0.05 were considered statistically significant. All statistical analyses were performed using the GraphPad Prism 7.0 software. Data were independently repeated for three times and presented as mean ± standard deviation.

## Results

### 19-HB inhibited NSCLC cells proliferation and suppressed colony formation

To investigate the in vitro antitumor effects of 19-HB, we treated NSCLC cells with concentration gradient of 19-HB for 24, 48, and 72 h and measured their cell viability using CCK-8 assay. The results showed that the exposure to 19-HB resulted in a significant decline in cell viability in a dose-dependent manner in NCI-H1299 and NCI-H838 cell lines, compared with the untreated group (*P* < 0.01, Fig. 1c and Additional file 1: Fig. S1). To further test the antitumor effects of 19-HB on other cancer cell types, liver cancer Huh7 and HepG2 cells, breast cancer MDA-MB-231 cells and lung cancer A549 cells were treated with 19-HB for indicated time. Similar results as H1299 and H838 were observed (Additional file 1: Fig. S2). Next, to assess the inhibitory effect on cell proliferation, which is essential in carcinogenesis, cell morphology and colony formation assay were adopted in a concentration gradient of 19-HB (0, 30, 60, 120 nM) after a 24-h treatment. In the results showed under the microscope, the cell counts significantly declined in the 19-HB-treated group compared with the control group, along with varying degrees of deformation and contraction (*P* < 0.01, Fig. 1b). Consistent with these, clonogenicity ability was significantly suppressed upon the treatment of 19-HB and led to a remarkable decrease in the colony formation ratio (*P* < 0.01, Fig. 1d). Taken together, the abovementioned results suggested that the treatment with 19-HB contributed to dose-dependent growth inhibition of NSCLC cells.

### 19-HB promoted apoptosis of NSCLC cells

**Fig. 2:**
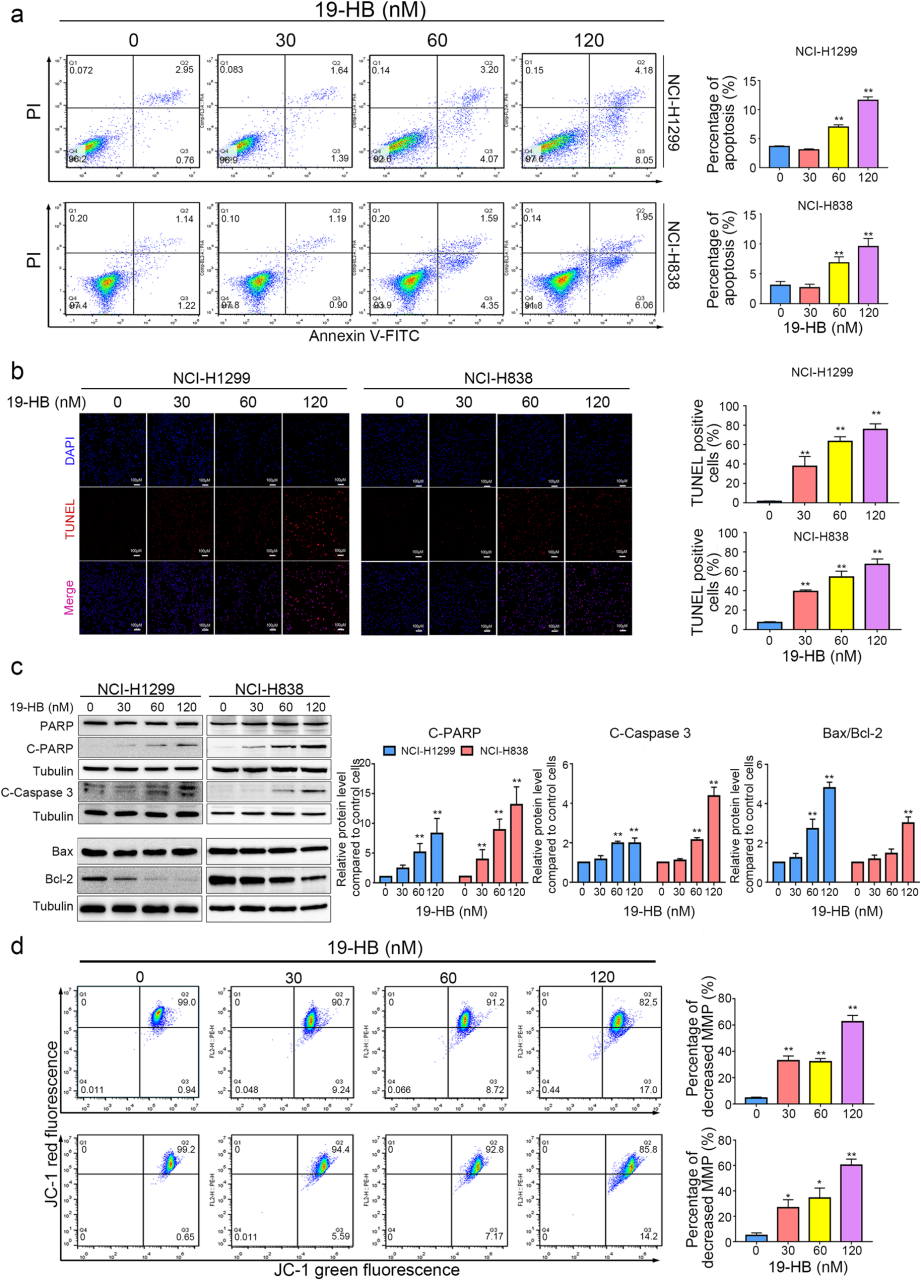
19-HB induced apoptosis in NSCLC cells. NCI-H1299 and NCI-H838 cells were treated for 24 h with the indicated concentrations of 19-HB. **a** Percentage of apoptotic cells in 19-HB-treated cells versus DMSO-treated cells determined using flow cytometry analysis. **b** Quantitative apoptosis changes detected by TUNEL assay. TUNEL (red) was used to mark fragmented DNA, while DAPI (blue) was used to label the cell nuclei. **c** Western blotting using cell extract to detect the expression of cleaved caspase-3, cleaved PARP, and Bax/Bcl-2. Tubulin was used as the loading control, and the quantitative data of the protein levels are shown. **d** The changes in mitochondrial membrane potential measured by a JC-1 mitochondrial membrane potential assay kit, and the mitochondrial membrane potential was quantitatively analyzed (*P < 0.05 vs. control, **P < 0.01 vs. control)

Apoptosis is a cell suicide program, which functions to restrict the cell counts and eliminate malignant cells. The apoptosis induction in target cells is a critical approach in antitumor therapies [19]. As described above, distinct morphological changes in NSCLC cells may be due to 19-HB-induced cell contraction and apoptosis. Moreover, to evaluate the apoptosis induced by 19-HB, we compared the apoptosis rate of 19-HB-treated and untreated groups using multiple methods, consisting of Annexin V/PI double staining assay, TUNEL assay, JC-1 staining, and western blotting. After 24 h of treatment with 19-HB, flow cytometry results showed that the apoptotic proportions of four concentrations (0, 30, 60, 120 nM) were 3.63 ± 0.12%, 3.07 ± 0.16%, 6.99 ± 0.37%, and 11.59 ± 0.60%, respectively, in NCI-H1299 cells, and 3.06 ± 0.65%, 2.66 ± 0.59%, 6.84 ± 0.99%, and 9.56 ± 1.35%, respectively, in NCI-H838 cells (mean ± SD, *P* < 0.01, Fig. 2a). Furthermore, TUNEL assay showed that DNA fragmentation was upregulated in a dose-dependent manner at 24 h, which also indicated the occurrence of cell apoptosis (Fig. 2b). Subsequently, we examined the levels of key proapoptotic genes (PARP and caspase-3) and mitochondrial apoptosis-related proteins (Bax/Bcl-2) in both NCI-H1299 and NCI-H838 cells by western blotting analysis. Interestingly, 19-HB markedly upregulated the expression of cleaved caspase-3, cleaved-PARP and the ratio of the Bax/Bcl-2 levels, compared with the control group (*P* < 0.01 vs. control, Fig. 2c). The necroptotic-related proteins, such as RIP, RIP3, and MLKL showed no significant changes in both cell lines (Additional file 1: Fig. S3). In conclusion, these results suggested that 19-HB reduced the proliferation of NSCLC cells through induction of apoptosis in vitro. We further inspected the mitochondrial membrane potential using JC-1 staining. Compared with the control group with 99.0% red fluorescence, the percentage gradually decreased to 90.7%, 91.2%, and 82.5% in the cells treated with the rising concentrations of 19-HB (*P* < 0.01 in NCI-H1299, *P* < 0.05 in NCI-H838); the ratio of green to red fluorescence increased as well (Fig. 2d). The above results indicated that 19-HB activated the mitochondrial apoptosis pathway in NSCLC cells.

### 19-HB inhibited migration and invasion of NSCLC cells

**Fig. 3:**
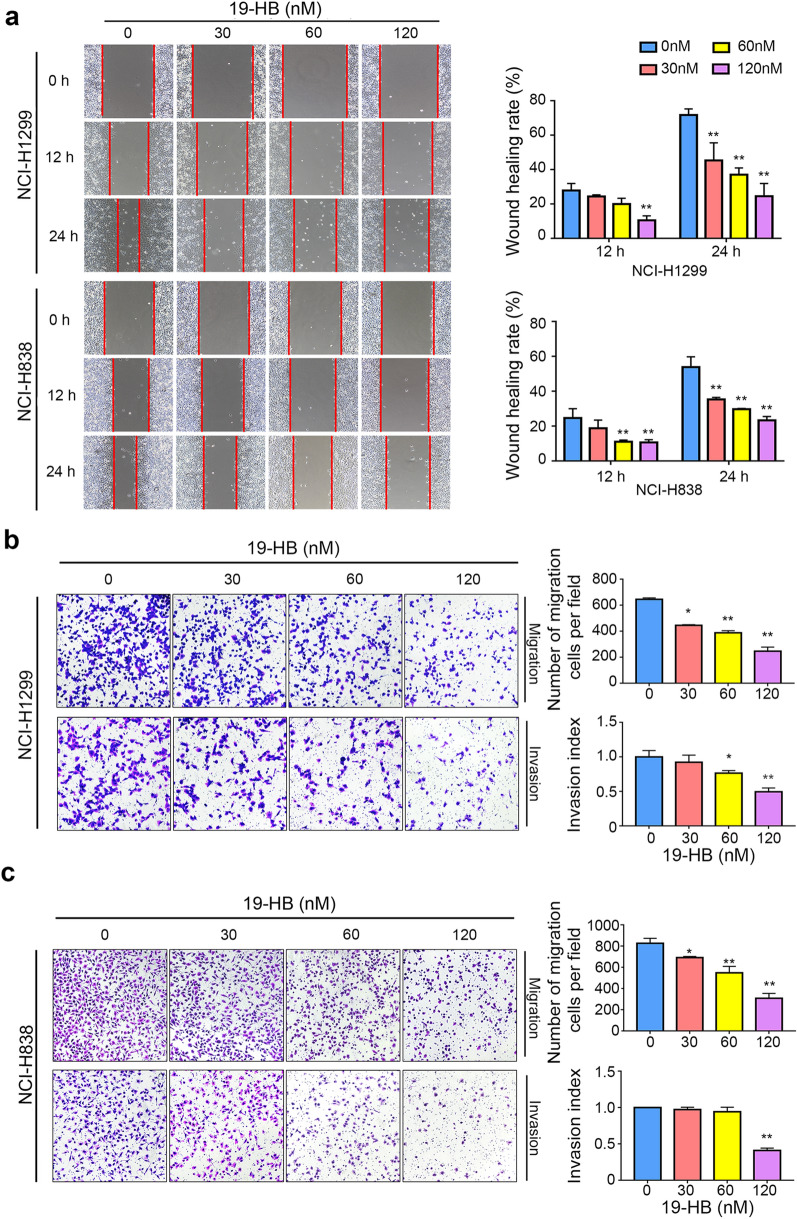
19-HB inhibited NSCLC cells migration and invasion. NCI-H1299 and NCI-H838 cells were treated for 24 h with the indicated concentrations of 19-HB. **a** Wound healing rate quantitatively analyzed at the indicated times (12 and 24 h after injury). **b**, **c** Migration and invasion of NCI-H1299 (**b**) and NCI-H838 (**c**) cells measured by transwell assay, and quantified by counting the migrating cells and calculating the invasion index in five randomly chosen high-power fields for each replicate (n = 3) (*P < 0.05 vs. control, **P < 0.01 vs. control)

19-HB repressed the mobility of NCI-H838 and NCI-H1299 cells as demonstrated in wound healing assay and transwell assay. Wound healing assay showed lower mobility of NSCLC cells compared with the untreated group (*P* < 0.01), and this effect positively correlated with the concentration of 19-HB (Fig. 3a). Moreover, migration and invasion abilities were also suppressed distinctly as shown in the transwell assay (*P* < 0.01, Fig. 3b, c). Taken together, we showed that 19-HB attenuated the mobility of NSCLC cells.

**Fig. 4:**
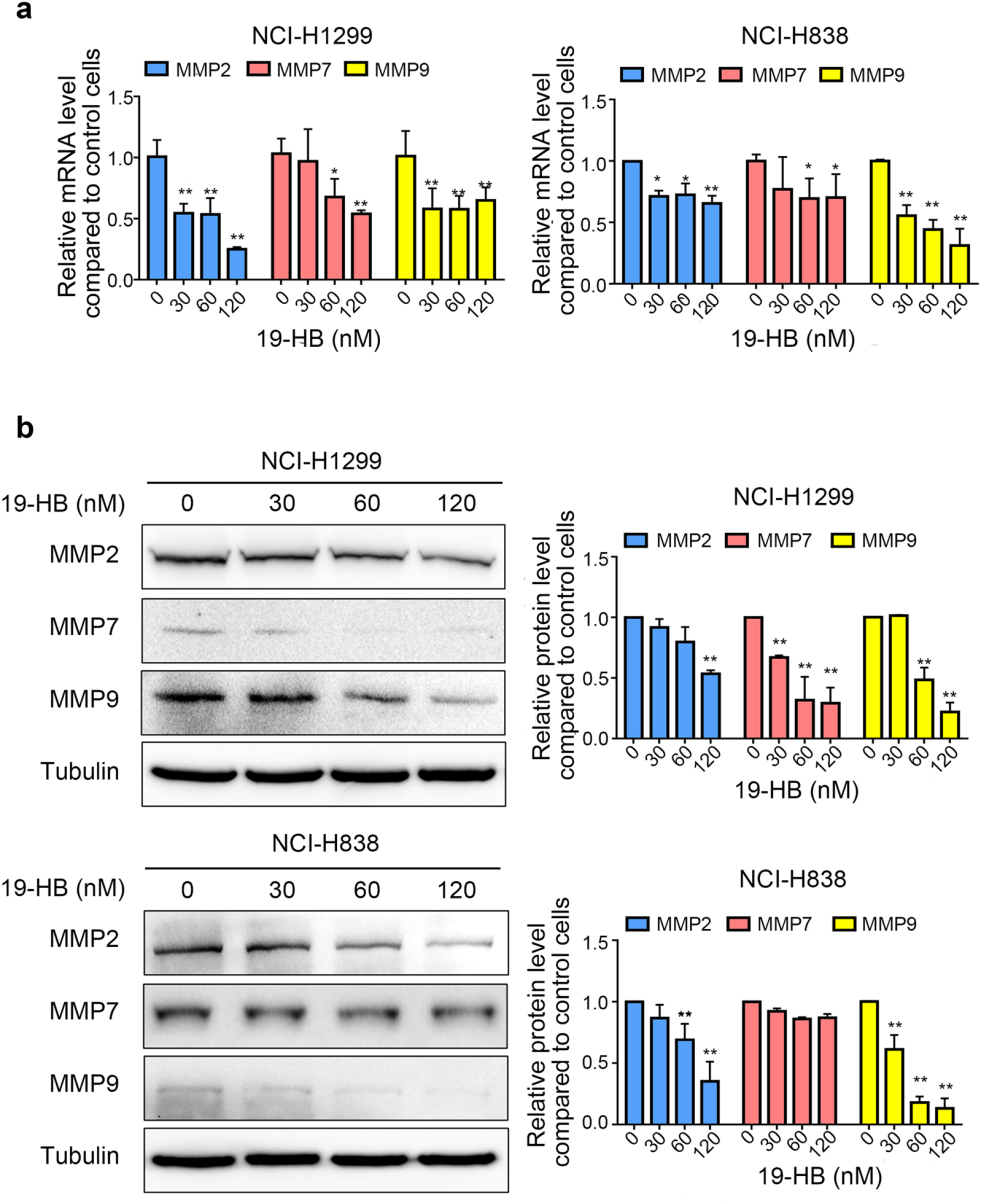
19-HB inhibited expression levels of MMPs in NSCLC cells. **a** mRNA expression of MMPs in NSCLC cell lines. **b** Protein levels of MMPs in NSCLC indicated by western blotting. Tubulin was used as the loading control, and the quantitative data of the protein levels are shown (*P < 0.05 vs. control, **P < 0.01 vs. control)

The crucial step for the migration and invasion processes is passing throughout the extracellular matrix; the proteolytic activity is required for this process, including MMP2, MMP9, and MMP7, where the first two selectively degrade the major components of the ECM, while the third enzyme participates in metastasis [20]. To determine whether 19-HB inhibits migration and invasion through regulating MMPs, we examined the mRNA and protein levels of MMP2, MMP7, and MMP9 in 19-HB-treated NSCLC cells. As shown in Fig. 4, the expression levels of MMPs decreased at both mRNA and protein levels. These data suggested that 19-HB inhibited the mobility of NSCLC cells by also downregulating the expression of MMPs.

**Fig. 5:**
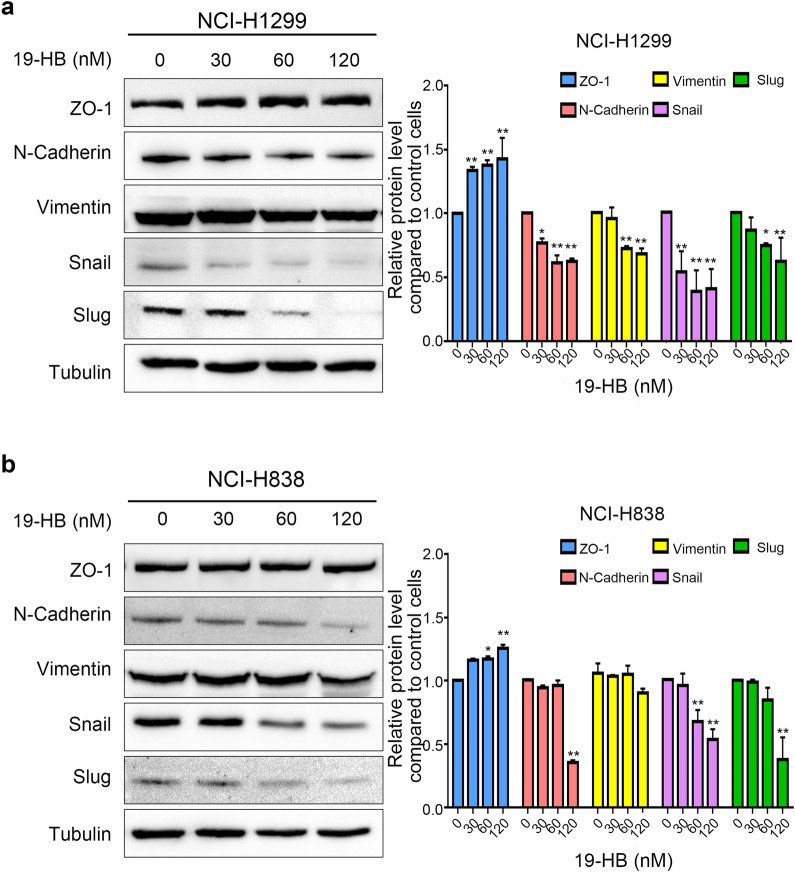
19-HB inhibited expression levels of EMT-associated factors in NSCLC cells. **a**, **b** Protein levels of EMT and relative protein expression in NCI-H1299 (**a**) and NCI-H838 (**b**) cells (*P < 0.05 vs. control, **P < 0.01 vs. control)

In addition, western blotting was used to measure the variations in epithelial–mesenchymal transition (EMT)-related molecules to assess the influence of 19-HB on EMT. As shown in Fig. 5, compared with the untreated group, the epithelial marker ZO-1 increased (*P* < 0.01), and the mesenchymal markers N-cadherin and Vimentin decreased. Since specific EMT-associated transcription factors (EMT-TFS) drive EMT process, we investigated whether the expression of EMT-TFS is regulated by 19-HB. The results showed that 19-HB suppressed the expression level of Snail and Slug (*P* < 0.01, Fig. 5a, b). A number of studies have shown that EMT is closely related to cancer metastasis. Therefore, our findings supported that the underlying mechanism of EMT suppression in 19-HB-treated NSCLC cells may be mediated by the inhibition of EMT-TFs.

### 19-HB inhibited the activation of the Wnt/β-catenin signaling pathway in NSCLC cells

**Fig. 6:**
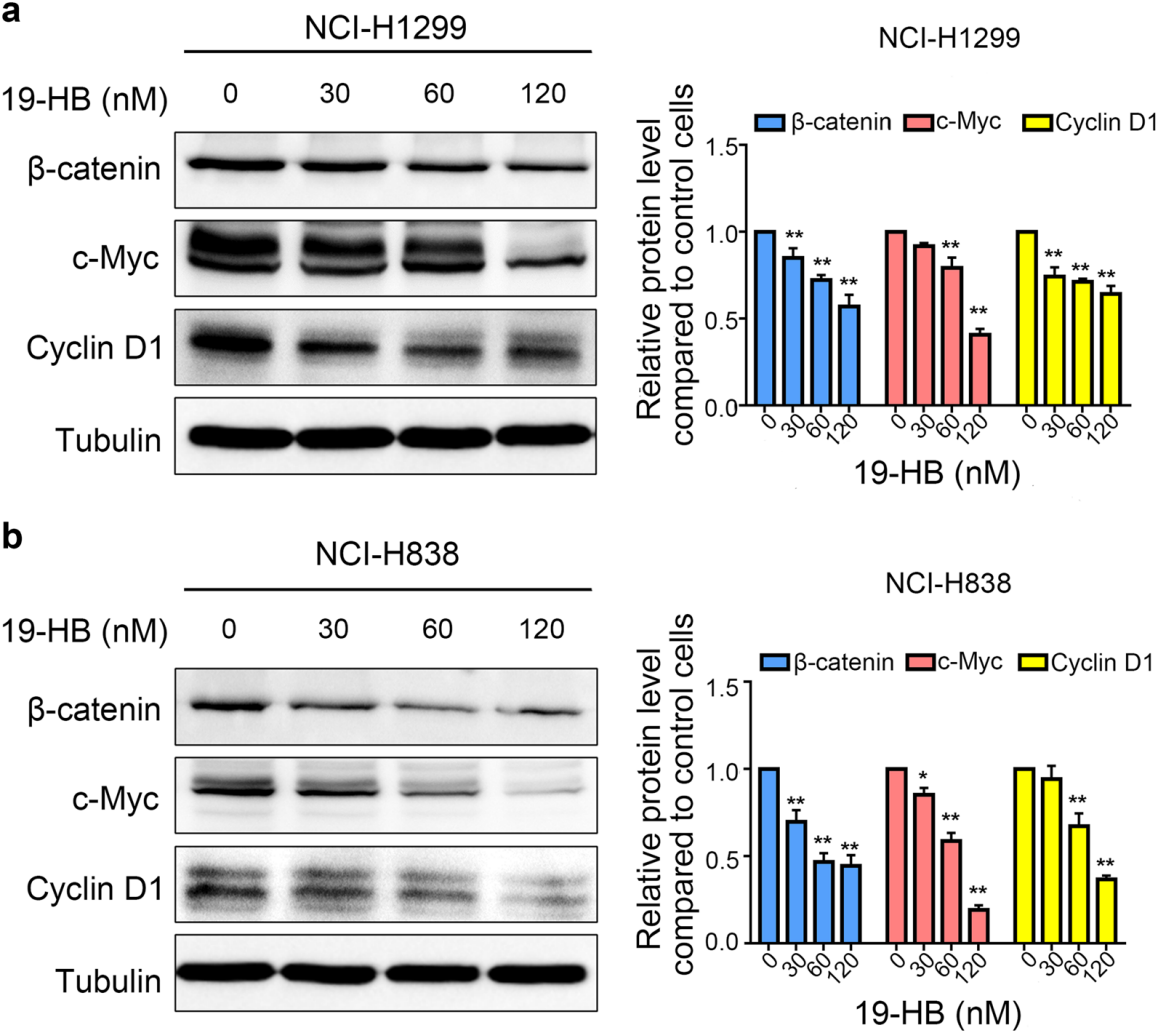
19-HB inhibited the Wnt/β-catenin signaling pathway. **a**, **b** Western blotting showed that 19-HB downregulated β-catenin, c-Myc, and Cyclin D1 in NCI-H1299 (**a**) and NCI-H838 (**b**) cells (*P < 0.05 vs. control, **P < 0.01 vs. control)

Since Wnt/β-catenin signaling pathway is important for regulating EMT process, we evaluated the expression levels of β-catenin, c-Myc, and Cyclin D1 by western blotting. All these proteins were key factors in Wnt/β-catenin signaling pathway. We found that 19-HB downregulated the protein expression of β-catenin (*P* < 0.05 vs. control), c-Myc (*P* < 0.05 vs. control), and Cyclin D1 (*P* < 0.01 vs. control, Fig. 6a, b). PDK1/AKT/MDM2 and NFκB pathways were also tested in 19-HB treated cells, the results showed that PDK1/AKT/MDM2 pathway related proteins including phosphorylated-PDK1, AKT, the ratio of phosphorylated-MDM2 and total MDM2, appeared no significant difference, the similar results were observed in NFκB pathway (Additional file 1: Fig. S4). Hence, 19-HB mainly inhibited NSCLC cells by suppressing Wnt/β-catenin signaling pathway.

### 19-HB inhibited the growth of NSCLC in vivo

**Fig. 7:**
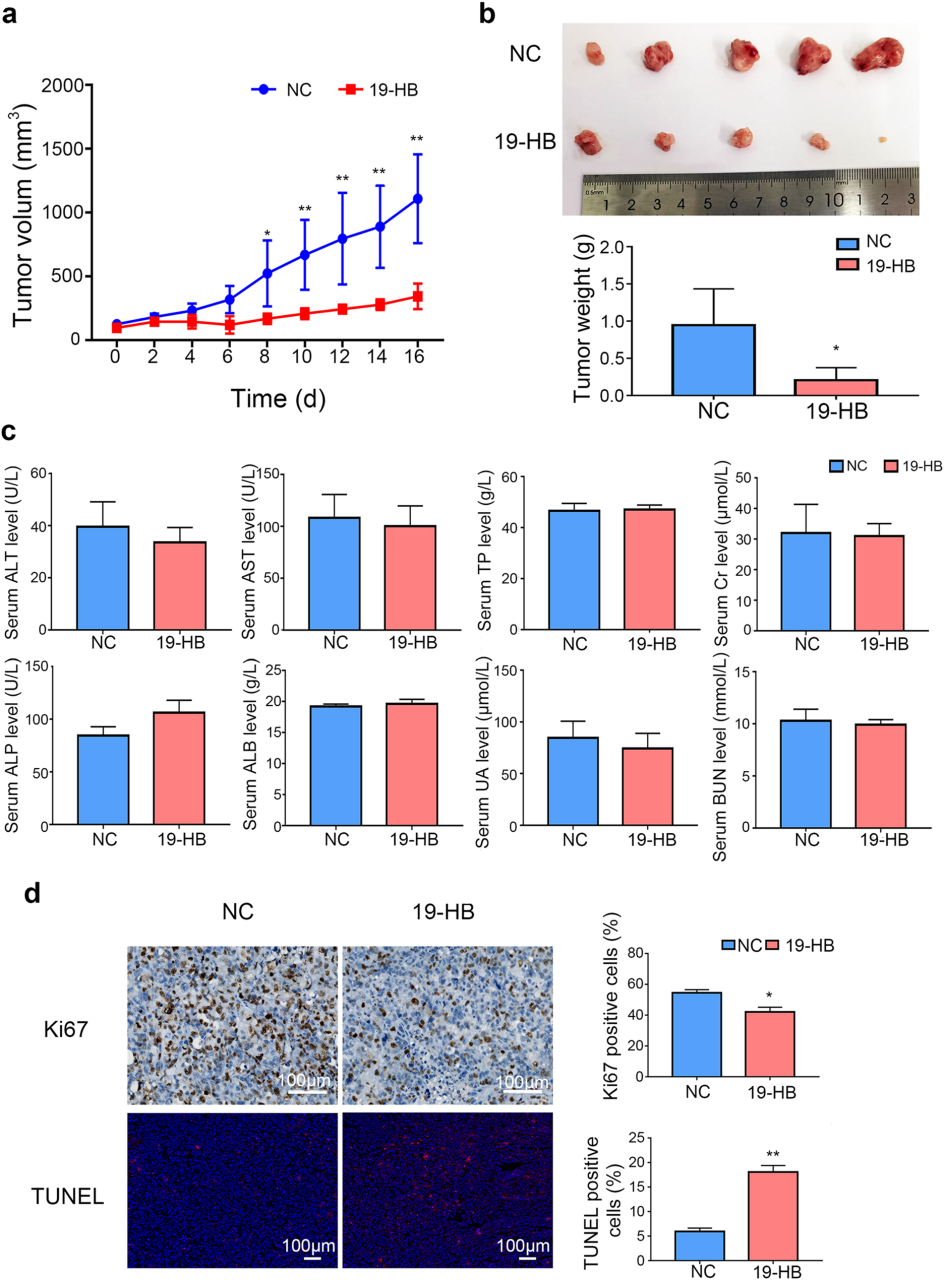
19-HB inhibited NSCLC growth in vivo. Xenograft Balb/c nude mice model was established with NCI-H1299 cells, and the mice were euthanized after completing the treatment. **a** Tumor volumes of tumor-bearing Bulb/c mice measured on the indicated days. **b** Tumor size and tumor weights measured. **c** Serum biochemical indexes to reflect the hepatorenal toxicity of 19-HB. **d** IHC staining for Ki67 and immunofluorescence staining for TUNEL assay performed in tumor mass, and the positive rate of Ki67 and TUNEL determined on IHC staining afterward (*P < 0.05 vs. control, **P < 0.01 vs. control)

Based on the in vitro data, we further investigated the in vivo antitumor ability of 19-HB in NCI-H1299 xenograft Balb/c nude mice. After administration of 19-HB to the mice, both the tumor weights (Fig. 7b) and tumor volume (Fig. 7a) in the treated group significantly decreased compared with those in the control group (*P* < 0.01). The results illustrated that 19-HB effectively inhibited the growth of NCI-H1299 tumor masses. Importantly, the nude mice had good tolerance to 19-HB with no obvious toxicity shown in biochemical analysis (Fig. 7c). In addition, IHC staining of Ki67 and immunofluorescence staining for TUNEL assay (Fig. 7d) showed reduced cell proliferation (*P* < 0.05) and increased apoptosis rate of the 19-HB group (*P* < 0.01). Our data suggested that 19-HB reduced the viability of NSCLC cells and inhibited the tumors growth in vivo.

## Discussion

Bufadienolides have shown significant drug potential for their antitumor activities, including suppressing cell proliferation, inducing apoptosis, inhibiting cancer angiogenesis, and regulating the immune response [21–26]. However, the molecular mechanism as to how these rare ingredients affect several biological functions both in vivo and in vitro remained elusive. 19-HB is a component of bufadienolides with significant antitumor activity, but few studies have focused on it or its biological activities. Thus, we evaluated the antitumor effects of 19-HB in NCI-H1299 and NCI-H838 cell lines.

We reported that 19-HB inhibited the proliferation of NSCLC cells. Subsequent experiments further confirmed that 19-HB showed extraordinary inhibitory effect on NSCLC migration and invasion in vitro along with a dose-dependent effect in promoting the apoptosis of NSCLC cells. Taking these results into account, we can hypothesize that 19-HB plays its antitumor activity by suppressing the cell proliferation, migration, and invasion of NSCLC cells.

Apoptosis is an important biological process that maintains homeostasis in organisms and cells. Apoptosis is a programmed cell death process; it shows chromatin condensation and nuclear fragmentation as the main characteristics, and it is accompanied by morphological changes and reduction in cellular volume [19]. Antitumor drugs mainly act by inhibiting cell proliferation and promoting apoptosis to kill tumor cells [27]. Both the intrinsic pathway and the extrinsic pathway mediates the cell apoptosis [28]. In the intrinsic apoptotic pathway, after the death signal is received, disruption of the mitochondrial membrane occurs subsequently, resulting in the release of cytochrome c from the mitochondria matrix into the cytosol and activation of caspase-3. Caspase-3 is one of the executor caspases for the apoptosis pathways. The activation of caspase-3 cleaves its substrate, PARP, into two fragments, thus triggering apoptosis through DNA fragmentation [29]. In addition, Bax—a member of the Bcl-2 family—also participates in the intrinsic apoptosis pathway [30]. Bax is generally present in the cytoplasm; after receiving the apoptotic signal, Bax relocates to the mitochondrial surface and forms a transmitochondrial membrane hole on the mitochondrial surface. Subsequently, the mitochondrial membrane potential decreases and membrane permeability increases, thus releasing apoptotic factors into the cytosol; this process can be measured by the ratio of Bcl-2 and Bax. In this study, we examined the mitochondrial membrane potential and the expression signature of apoptosis-related proteins in 19-HB-treated NSCLC cells. We also observed the necroptosis in 19-HB-treated cells, necroptotic-related proteins, such as RIP, MLKL, only showed a slightly changed, but did not achieve statistically significant. Our data indicated that 19-HB reduced the mitochondrial transmembrane potential, activated cleaved caspase-3, and cleaved PARP, ultimately leading to apoptosis.

Metastasis of malignant tumors is the most important cause of patient death and is the key problem that needs to be overcome during tumor treatment. Previous studies showed that the expression levels of MMP2, MMP7 and MMP9 is positively correlated with the plasticity of tumor cells, such as invasion depth, metastasis distance, and vascular permeability [31]. Therefore, we further explored the changes in EMT-related proteins in 19-HB-treated NSCLC cells; the results showed that 19-HB decreased the expression of the mesenchymal markers N-cadherin and Vimentin, while the epithelial marker ZO-1 was increased. Similarly, 19-HB suppressed the function of the transcription factors of EMT. EMT is one the most important biological behaviors in cancer, during EMT process, tumor cells acquire mesenchymal cell properties and lose their epithelial characteristics, which subsequently leads to high metastatic potential [32]. Therefore, 19-HB may inhibit tumor cell migration and invasion by regulating EMT signals. Moreover, we screened the leading signaling pathways that regulate EMT, such as the TGF-β, Notch, Wnt/β-catenin, PI3K/AKT, and ERK pathways [33], by western blotting; the results showed that 19-HB negatively regulated the expression of β-catenin, Cyclin D1, and c-Myc, the known targets of the Wnt/β-catenin signaling pathway [34–37]. The aberrant Wnt/β-catenin signaling pathway is known to play crucial roles in tumorigenesis and remains a hot target for anti-cancer drug development [38]. Therefore, our findings suggested that the antitumor effects of 19-HB in NSCLC cells can be mediated through suppression of Wnt/β-catenin signaling pathway.

The application of actionable TCM compounds in NSCLC opens the way for clinical treatment. We identified a monomer from the skin of toads, 19-HB, with good antitumor activity; we also clarified its primary mechanism of attenuating progression of NSCLC. However, some improvements are still needed to alleviate the untoward effects and improve the efficacy of first-line therapies for lung cancer; in that context, we foresee that the explorations of combination therapies with 19-HB could answer this question.

## Conclusions

Our study indicated that 19-HB inhibited proliferation of NSCLC cells, promoted cell apoptosis, and inhibited cell mobility. These effects were achieved by suppressing the expression of EMT-related markers and inhibiting the activity of the Wnt/β-catenin signaling pathway. Thus, 19-HB may be a potential antitumor agent for NSCLC.

## Supplementary Information

**Additional file 1: Fig. S1. **19-HB inhibited NSCLC cell growth. **Fig. S2.** 19-HB inhibited cell proliferation in certain cancer cell lines. **Fig. S3.** 19-HB had little effects of necroptosis in NSCLC cells. **Fig. S4.** 19-HB inhibited tumor cell growth not through the PDK1/AKT/MDM2 and NFκB signaling pathways.

## Data Availability

The data used during the current study are available from the corresponding author on reasonable request.

## Abbreviations

19-HB: 19-Hydroxybufalin
ALB: Albumin
ALP: Alkaline phosphatase
ALT: Alanine aminotransferase
AST: Aspartate transaminase
Bax: Bcl-associated x protein
BCA: Bicinchoninic acid
Bcl-2: B cell lymphoma/leukemia-2
BSA: Bovine serum albumin
BUN: Blood urea nitrogen
Caspase: Cysteine-containing aspartate specific protease
CR: Creatinine
ECM: Extracellular matrix
EMT: Epithelial-to-mesenchymal transition
IHC: Immunohistochemistry
MMP: Matrix metalloproteinase
NSCLC: Non-small cell lung cancer
OD: Optical density
RT‐qPCR: Real-time quantitative polymerase chain reaction
TP: Total protein
TUNEL: Terminal deoxynucleotidyl mediated dUTP nick end transferase labeling
UA: Uric acid
