# Supplementary material for: 19-Hydroxybufalin inhibits non-small cell lung cancer cell proliferation and promotes cell apoptosis via the Wnt/β-catenin pathway
Source: Exp Hematol Oncol. 2021 Oct 25;10:48. doi: 10.1186/s40164-021-00243-0 (PMC8543904; doi:10.1186/s40164-021-00243-0)
Supplement: Supplementary file 1 — Additional file 1: Fig. S1. 19-HB inhibited NSCLC cell growth. Fig. S2. 19-HB inhibited cell proliferation in certain cancer cell lines. Fig. S3. 19-HB had little effects of necroptosis in NSCLC cells. Fig. S4. 19-HB inhibited tumor cell growth not through the PDK1/AKT/MDM2 and NFκB signaling pathways. [file 40164_2021_243_MOESM1_ESM.docx]

Additional file 1 of 19-Hydroxybufalin inhibits non-small cell lung cancer cell proliferation and promotes cell apoptosis via the Wnt/β-catenin pathway

Wei Yu^1,2^, Xiao Zhang^2^, Wei Zhang^2^, Minggang Xiong^2^, Yuhan Lin^2^, Ming Chang^2^, Lin Xu^1^, Yi Lu^2,3*^, Yun Liu^1*^ Jian Zhang^2,3*^


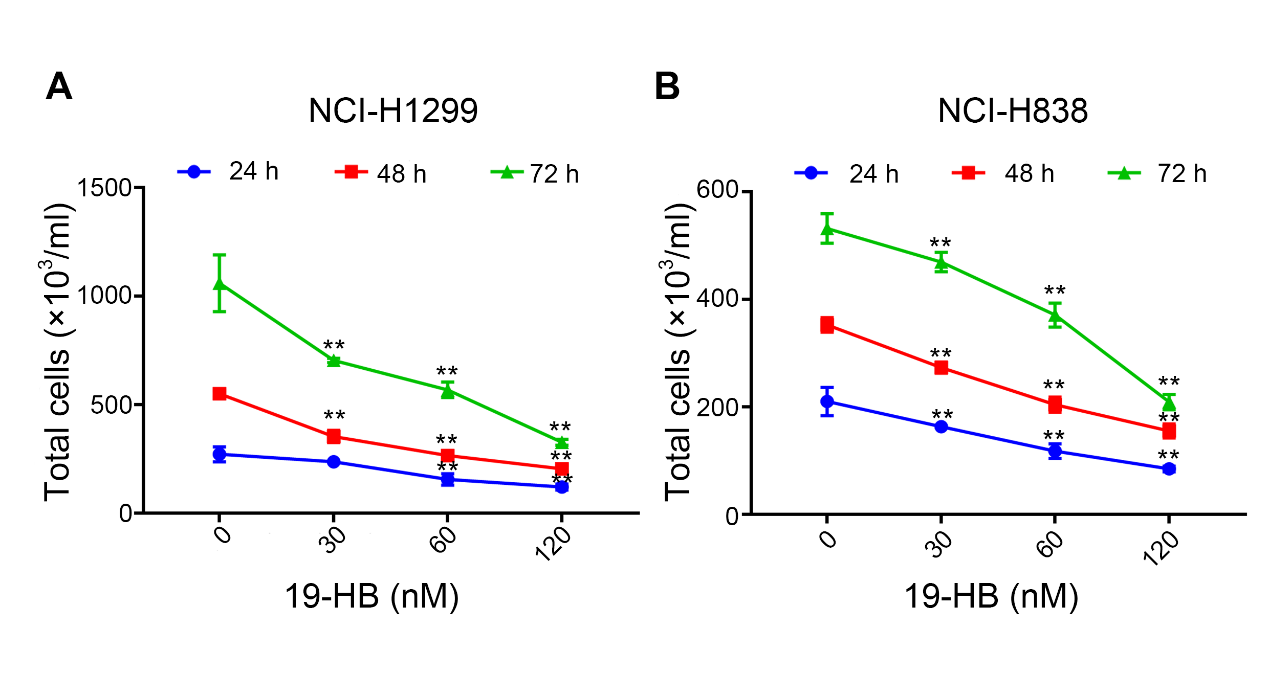


**Fig. S1: 19-HB inhibited NSCLC cell growth. (**A) NCI-H1299 cells, (B) NCI-H838 cells. The cells were seeded in 6-well plate and treated with 19-HB. Cell viability was measured by cell counting. The data are presented as the mean ± SD of three parallel tests (**P* < 0.05 *vs.* control, ***P* < 0.01 *vs.* control).


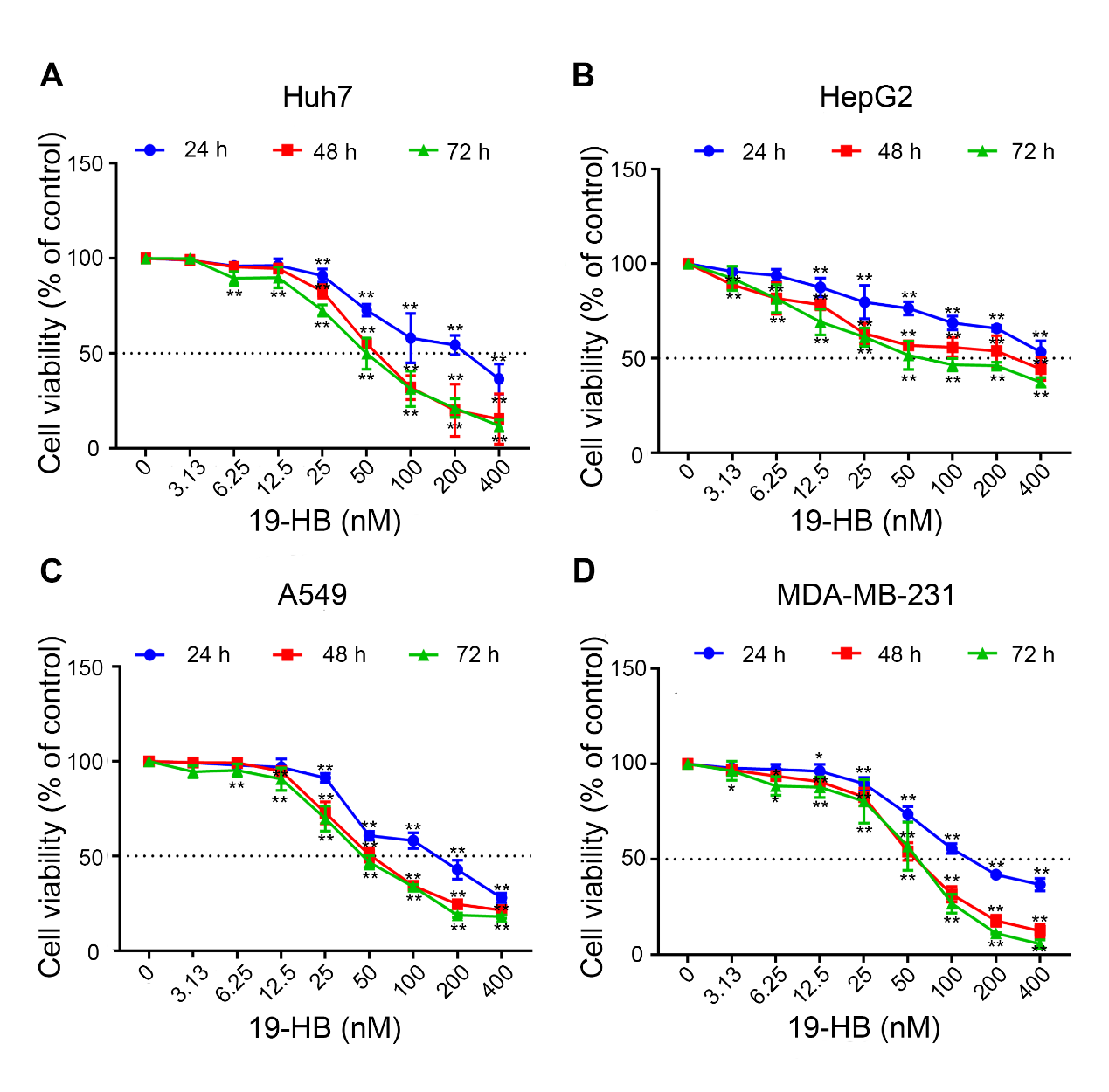


**Fig. S2: 19-HB inhibited cell proliferation in certain cancer cell lines. (**A) Liver cancer Huh7 cells, (B) Liver cancer HepG2 cells, (C) Lung cancer A549 cells, (D) Breast cancer MDA-MB-231 cells. Cell viability was determined by a CCK-8 assay at 24, 48, and 72 hours after treatment. The data are presented as the mean ± SD of three parallel tests (**P* < 0.05 *vs.* control, ***P* < 0.01 *vs.* control).


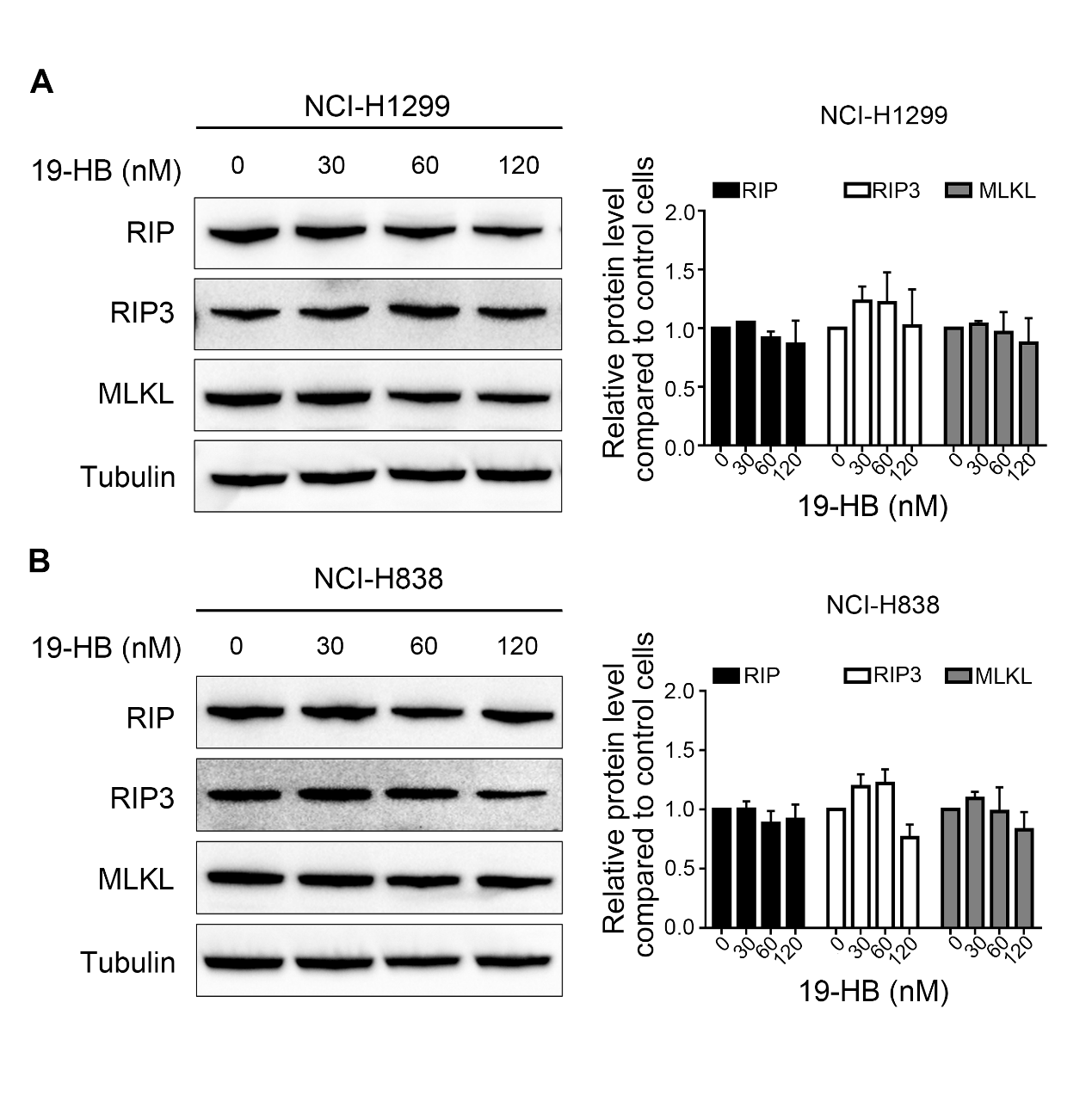


**Fig. S3: 19-HB had little effects of necroptosis in NSCLC cells.** (A) NCI-H1299, (B) NCI-H838 cells were treated for 24 hours with the indicated concentrations of 19-HB. Western blotting using cell extract to detect the expression of necroptotic-related proteins, such as RIP, RIP3 and MLKL. Tubulin was used as the loading control, and the quantitative data of the protein levels are shown.


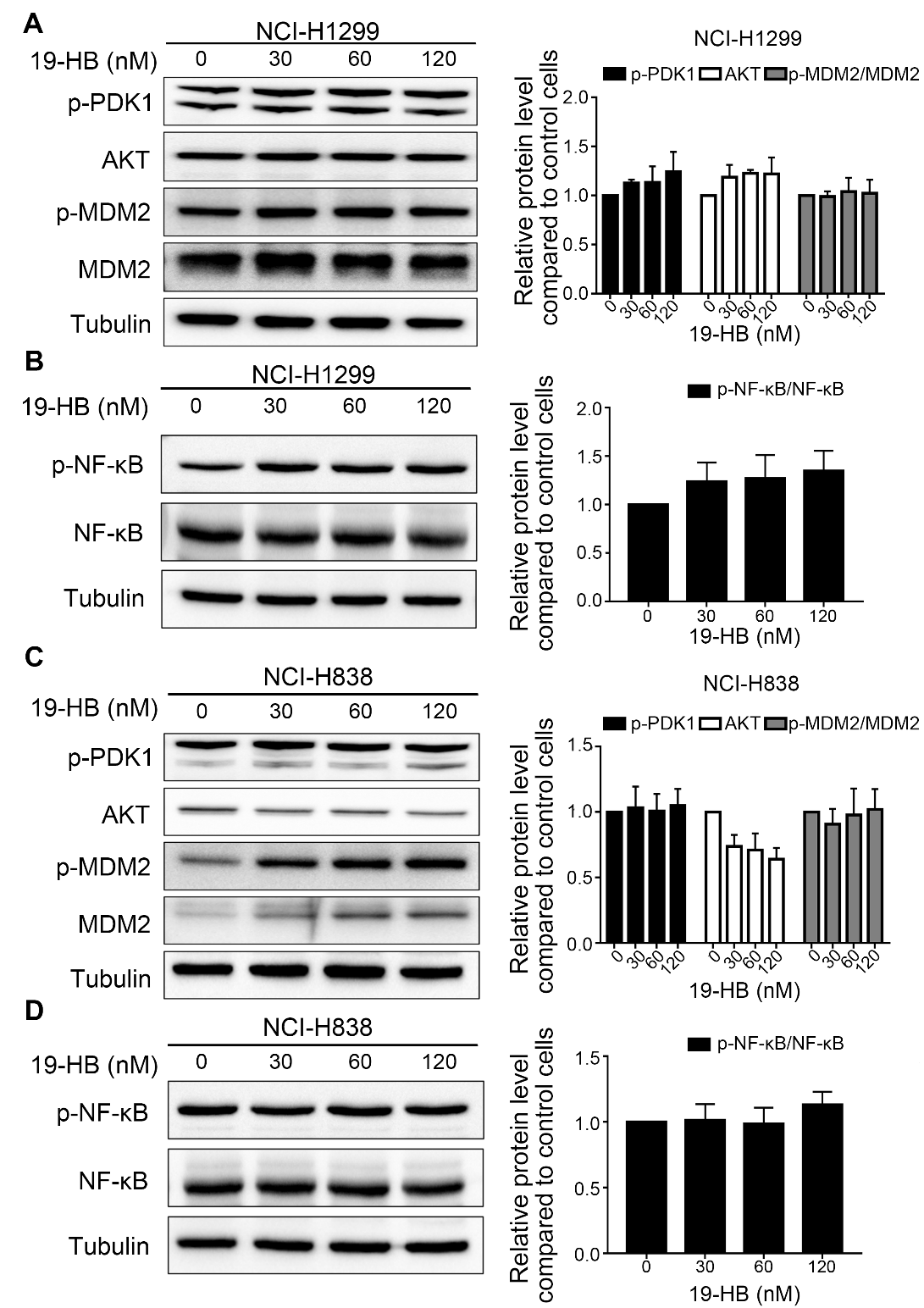


**Fig. S4: 19-HB inhibited tumor cell growth not through the PDK1/AKT/MDM2 and NFκB signaling pathways.** (A,B) NCI-H1299, (C,D) NCI-H838. Western blotting showed that PDK1/AKT/MDM2 pathway related proteins including phosphorylated-PDK1, AKT, the ratio of phosphorylated-MDM2 and total MDM2, appeared no significant difference, the similar results were observed in NFκB pathway.
